# SARS-CoV-2 RNA in Wastewater Was Highly Correlated With the Number of COVID-19 Cases During the Fourth and Fifth Pandemic Wave in Kobe City, Japan

**DOI:** 10.3389/fmicb.2022.892447

**Published:** 2022-06-09

**Authors:** Yoshihiko Tanimoto, Erika Ito, Sonoko Miyamoto, Ai Mori, Ryohei Nomoto, Noriko Nakanishi, Naohiro Oka, Takao Morimoto, Tomotada Iwamoto

**Affiliations:** ^1^Department of Infectious Diseases, Kobe Institute of Health, Kobe City, Japan; ^2^Planning Division, Sewage Works Department, Public Construction Projects Bureau, Kobe City, Japan

**Keywords:** COVID-19, SARS-CoV-2, sewage, wastewater, environmental surveillance

## Abstract

Severe acute respiratory syndrome coronavirus 2 (SARS-CoV-2), the cause of the current coronavirus disease 2019 (COVID-19) pandemic and associated respiratory infections, has been detected in the feces of patients. Therefore, determining SARS-CoV-2 RNA levels in sewage may help to predict the number of infected people within the area. In this study, we quantified SARS-CoV-2 RNA copy number using reverse transcription quantitative real-time PCR with primers and probes targeting the N gene, which allows the detection of both wild-type and variant strain of SARS-CoV-2 in sewage samples from two wastewater treatment plants (WWTPs) in Kobe City, Japan, during the fourth and fifth pandemic waves of COVID-19 between February 2021 and October 2021. The wastewater samples were concentrated *via* centrifugation, yielding a pelleted solid fraction and a supernatant, which was subjected to polyethylene glycol (PEG) precipitation. The SARS-CoV-2 RNA was significantly and frequently detected in the solid fraction than in the PEG-precipitated fraction. In addition, the copy number in the solid fraction was highly correlated with the number of COVID-19 cases in the WWTP basin (WWTP-A: *r* = 0.8205, *p* < 0.001; WWTP-B: *r* = 0.8482, *p* < 0.001). The limit of capturing COVID-19 cases per 100,000 people was 0.75 cases in WWTP-A and 1.20 cases in WWTP-B, respectively. Quantitative studies of RNA in sewage can be useful for administrative purposes related to public health, including issuing warnings and implementing preventive measures within sewage basins.

## Introduction

The coronavirus disease 2019 (COVID-19) outbreak, caused by the severe acute respiratory syndrome coronavirus 2 (SARS-CoV-2), started in December 2019 and then spread worldwide in 2020 (Huang et al., 2020). In Japan, the COVID-19 pandemic can be divided into five major waves as of December 2021 (Worldometer, 2020). COVID-19 infections occurred mainly due to alpha and delta variants in the fourth (March to June 2021) and fifth (August to October 2021) waves, respectively (Hodcroft, 2021). SARS-CoV-2 mainly causes symptoms of upper respiratory tract infections, but it may also cause severe pneumonia including acute respiratory distress syndrome (ARDS; Wu et al., 2020). Because SARS-CoV-2 can also infect the digestive organs, high levels of the virus can be detected in the feces of infected individuals (Lescure et al., 2020). Stool samples from 48.1% of patients tested positive for SARS-CoV-2 RNA and 70.3% of these patients had stool viral RNA that remained positive even when respiratory specimens were negative, as shown in a meta-analysis study (Cheung et al., 2020). Thus, SARS-CoV-2 is not only a case of respiratory distress, but is also one of the most important causative agents of human gastroenteritis.

Since municipal wastewater contains microorganisms derived from human feces, the concentration of pathogens in sewage is affected by infectious disease epidemics, mainly gastroenteritis, in the watershed population. For example, a previous study showed that the RNA copy number of human gastroenteric norovirus in sewage was significantly related to the number of gastroenteritis cases in the wastewater treatment plant (WWTP) basin (Kazama et al., 2017). In addition, hepatitis E virus was detectable in raw sewage when 1%–4% of residents in a WWTP basin were infected (Miura et al., 2016). Monitoring the prevalence of SARS-CoV-2 in wastewater is also considered to be an effective approach for predicting the COVID-19 epidemic following the detection of SARS-CoV-2 RNA in sewage in several countries (Ahmed et al., 2020a; La Rosa et al., 2020b; Wang et al., 2020; Wurtzer et al., 2020, 2022; Albastaki et al., 2021; Wehrendt et al., 2021; Kevill et al., 2022). In some of these studies, the RNA copy number of SARS-CoV-2 in the sewage was correlated with the number of COVID-19 clinical cases (Medema et al., 2020a; Carrillo-Reyes et al., 2021; Nagarkar et al., 2021; Street et al., 2021; Wurtz et al., 2021; Monteiro et al., 2022). In Japan, SARS-CoV-2 RNA was first detected in secondary treated wastewater before chlorination at a WWTP in Yamanashi Prefecture in April 2020 (Haramoto et al., 2020). Although experiments to detect SARS-CoV-2 RNA have been carried out on wastewater in other regions in Japan for 1–3 months (Hata et al., 2021; Kitamura et al., 2021; Nagashima et al., 2021; Torii et al., 2021), few studies have reported the relationship between the number of COVID-19 cases and the amount of RNA detected with long-term monitoring.

Owing to the low concentration of pathogenic viruses in wastewater, a method for concentrating and detecting these viruses is necessary (Haramoto et al., 2018). Various methods aimed at concentrating RNA in environmental samples to improve detection have been evaluated (Ahmed et al., 2020b; Weidhaas et al., 2021). Among them, electronegative membrane adsorption, polyethylene glycol (PEG) precipitation, and ultrafiltration are frequently used to detect non-enveloped viruses such as poliovirus and norovirus (World Health Organization, 2003; Kazama et al., 2017). Several studies have employed these methods to enrich SARS-CoV-2 RNA (Foladori et al., 2020; La Rosa et al., 2020a; Sangkham, 2021); however, since SARS-CoV-2 is an enveloped virus, the concentration efficiency of the virus differs from that of non-enveloped viruses. Recently, comparative studies of extraction methods showed that SARS-CoV-2 RNA was more abundantly detected in the solid fraction of sewage samples, i.e., the pellet obtained by centrifugation of sewage samples (Kitamura et al., 2021; Westhaus et al., 2021). On the other hand, another study reported that approximately 90% of the SARS-CoV-2 RNA was present in the liquid phase of the influent wastewater (Weidhaas et al., 2021). The assessment of RNA concentration from sewage solids is important, even though solid residues are largely removed in studies on wastewater treatment.

Evaluating the efficiency of the process after wastewater concentration requires the use of a control virus. Pepper mild mottle virus (PMMoV) is the most abundant virus in human feces (Zhang et al., 2006), owing to which it can be easily quantified without spiking in a wastewater sample. High concentrations of PMMoV have been detected in water environment (Rosario et al., 2009; Haramoto et al., 2013; Hughes et al., 2017), and this virus has been used as an internal control for virus detection in wastewater in several studies (D’Aoust et al., 2021; Gerrity et al., 2021; Rosiles-González et al., 2021).

In the present study, we examined the pelleted solid fraction and the product of PEG precipitation of the supernatant fraction of wastewater samples for SARS-CoV-2 RNA. Wastewater was collected once a week from two WWTPs in Kobe, Japan, during the fourth and fifth pandemic waves of COVID-19, and the relationship between the SARS-CoV-2 RNA copy number in the two sample types was correlated with the reported number of COVID-19 cases in the corresponding sewage basin.

## Materials and Methods

### Sample Collection

Influent wastewater samples were collected once a week from 24 February to 27 October 2021 at WWTP-A (*n* = 36) and WWTP-B (*n* = 36) in Kobe City, Japan. The samples were grabbed from the influent, which comprised wastewater before treatment at the WWTPs. All sampling was performed at a fixed time every Wednesday, except on May 6 (WWTP-A and WWTP-B) and August 12 (WWTP-B), in which samples were collected on a Thursday. The samples were collected in sterile plastic bottles and kept frozen at −20°C until analysis. As of December 2021, the city had 1,515,907 inhabitants, of which 98.7% were covered by six WWTPs. WWTP-A and WWTP-B covered 51.5% of the population, received 51.3% of the total wastewater, and treated a total flow of 364,100 m^3^ per day. The amounts of rainfall (mm/day) and influent flow (m^3^/day) were measured as routine work at each WWTP.

### RNA Extraction

Viral RNA was extracted from each sewage sample after centrifugation, to produce a solid fraction, and after PEG precipitation of the supernatant, to produce a PEG-precipitated fraction, following the procedures of previous studies with minor modifications (Jones and Johns, 2009; Kitamura et al., 2021). Specifically, 160 ml of each sample was divided equally into four aliquots (40 ml each) held in 50 ml tubes and centrifuged at 10,000 × *g* for 30 min. RNA was extracted from the resulting pellet (solid fraction sample) using the NucleoBond RNA Soil kit (Macherey-Nagel, Düren, Germany) following the manufacturer’s instructions. Meanwhile, the entire supernatant was precipitated using PEG 8000 (final concentration 10%; Promega, Madison, WI, United States) and NaCl (final concentration 1 M; Wako, Tokyo, Japan) by incubating at 4°C overnight with gentle rotation. After centrifugation at 10,000 × *g* for 60 min, the precipitate was resuspended in 500 μl of phosphate buffer solution (pH 7.0, 0.067 mol/L; Nacalai Tesque, Kyoto, Japan). RNA was extracted from 140 μl of the PEG-precipitated suspension using a QIAamp Viral RNA Kit (Qiagen, Hilden, Germany). RNA was also extracted from 140 μl of raw unconcentrated sewage samples using a QIAamp Viral RNA Kit (Qiagen).

### Reverse Transcription-Quantitative PCR

To quantify viral RNA in the samples, reverse transcription-quantitative PCR (RT-qPCR) was performed using the Thermal Cycler Dice Real Time System III (Takara Bio, Shiga, Japan). SARS-CoV-2 RNA was quantified in the solid fraction, the PEG-precipitated sample, and unconcentrated sewage samples using the TaqMan Fast Virus 1-Step Master Mix (Applied Biosystems, Foster City, CA, United States) with combination of CDC 2019-nCoV_N1 and CDC 2019-nCoV_N2 primers and probes, which can be used to detect both the wild type and variant strains. The primer sequences used are described in Supplementary Table S1. Thermal cycling conditions included an initial incubation at 50°C for 5 min and initial denaturation at 95°C for 20 s, followed by 45 cycles of denaturation at 95°C for 3 s and annealing and extension at 60°C for 30 s, as per the manufacturer’s instructions. PMMoV RNA was also quantified in the same samples using the One Step PrimeScript III RT-qPCR Mix (Takara Bio). Thermal cycling conditions for PMMoV included an initial incubation at 52°C for 5 min and initial denaturation at 95°C for 10 s, followed by 45 cycles of denaturation at 95°C for 5 s and annealing and extension at 60°C for 30 s, as per the manufacturer’s instructions. All RT-qPCR analyses included both positive (standard DNA/RNA) and negative (water) controls. The analysis of SARS-CoV-2 samples was performed in duplicate; samples in which only one of the reactions showed a positive amplification were considered as negative overall. To obtain a standard curve for each assay, 10-fold dilution series of a standard plasmid DNA (PMMoV; 5 × 10^3^–5 × 10^6^; Haramoto et al., 2013) or RNA (2.5 × 10^0^ and 5 × 10^0^–5 × 10^4^; SARS-CoV-2 RNA positive control; Takara Bio) solutions were prepared for each assay. RNA copy numbers were calculated from the Ct values using the standard curves. The limit of quantification for SARS-CoV-2 was set at 2.5 copies/reaction (Supplementary Figure S1).

### Calculation of Viral RNA Copy Number and Recovery Rate

The copy number of the viral RNA calculated using RT-qPCR was corrected to copy/L as previously described (Qiu et al., 2022), as follows:


$$\begin{array}{l} \mathrm{RNA}\;\mathrm{copy number}\;\left(\mathrm{copy}/L\right)\\ =\left(\begin{array}{l} \mathrm{RNA}\;\mathrm{copy number}\;\left(\mathrm{copy}/\mathrm{reaction}\right)\\ \times \frac{V_{\mathrm{extracted}\;\mathrm{RNA}}}{V_{\mathrm{RNA}\;\mathrm{in each}\;\mathrm{PCR}\;\mathrm{reaction}}}\\ \times \frac{V_{\mathrm{wastewater concentrate}}}{V_{\mathrm{wastewater concentrate for}\;\mathrm{RNA}\;\mathrm{extraction}}}\times \frac{1000}{V_{\mathrm{intial wastewater}}} \end{array}\right) \end{array}$$

where V_extracted RNA_ is the total volume of the extracted RNA, V_RNA in each PCR reaction_ is the volume of RNA assayed in a RT-PCR reaction, V_wastewater concentrate_ is the sample volume after concentration, V_wastewater concentrate for RNA extractions_ is the volume of wastewater concentrate used for RNA extraction, and V_initial wastewater_ is the volume of initial wastewater sample processed.

Meanwhile, the recovery rate (%) was calculated using PMMoV quantitative value as follows:


$$\begin{array}{l} \mathrm{Recovery rate}\;\left(\%\right)\\ =\frac{PMMoV\;RNA\;copy\;number\;of\;concentrated\;sample\;\left(copy/L\right)}{PMMoV\;RNA\;copy\;number\;of\;unconcentrated\;sample\;\left(copy/L\right)}\\ \times 100 \end{array}$$

### Statistical Analysis

The daily newly reported number of COVID-19 cases was obtained from the Coronavirus Infection Status Report, which is a public database from Kobe City (2021). This database includes the symptomatic cases reported by hospitals and private COVID-19 test centers, as well as asymptomatic cases tested for contact tracing conducted by Public Health Management Center, Kobe City. The number of COVID-19 cases in each investigated basin of the WWTPs were provided by the Public Health Division, Public Health Management Center, Kobe City. Statistical analyses were performed using GraphPad Prism 8 software (GraphPad Prism Software, San Diego, CA, United States). The slope, intercept, and coefficient of determination (*R^2^*) values between standard RNA and Ct value, and case numbers and RNA copy numbers were calculated using linear regression; in the latter case, regression through the origin was used. The detection frequency of SARS-CoV-2 between the solid and liquid fractions was assessed using Fisher’s extract test. The Mann–Whitney U test was used to compare RNA copy numbers of the different sewage treatment samples. To compare case numbers and RNA copy numbers, and amount of rainfall/influent flow and RNA copy numbers, the correlation coefficient (*r*) was calculated using Spearman’s correlation coefficient.

## Results

### Comparison of the Solid Fraction and PEG-Precipitated Sewage Samples

**Figure 1:**
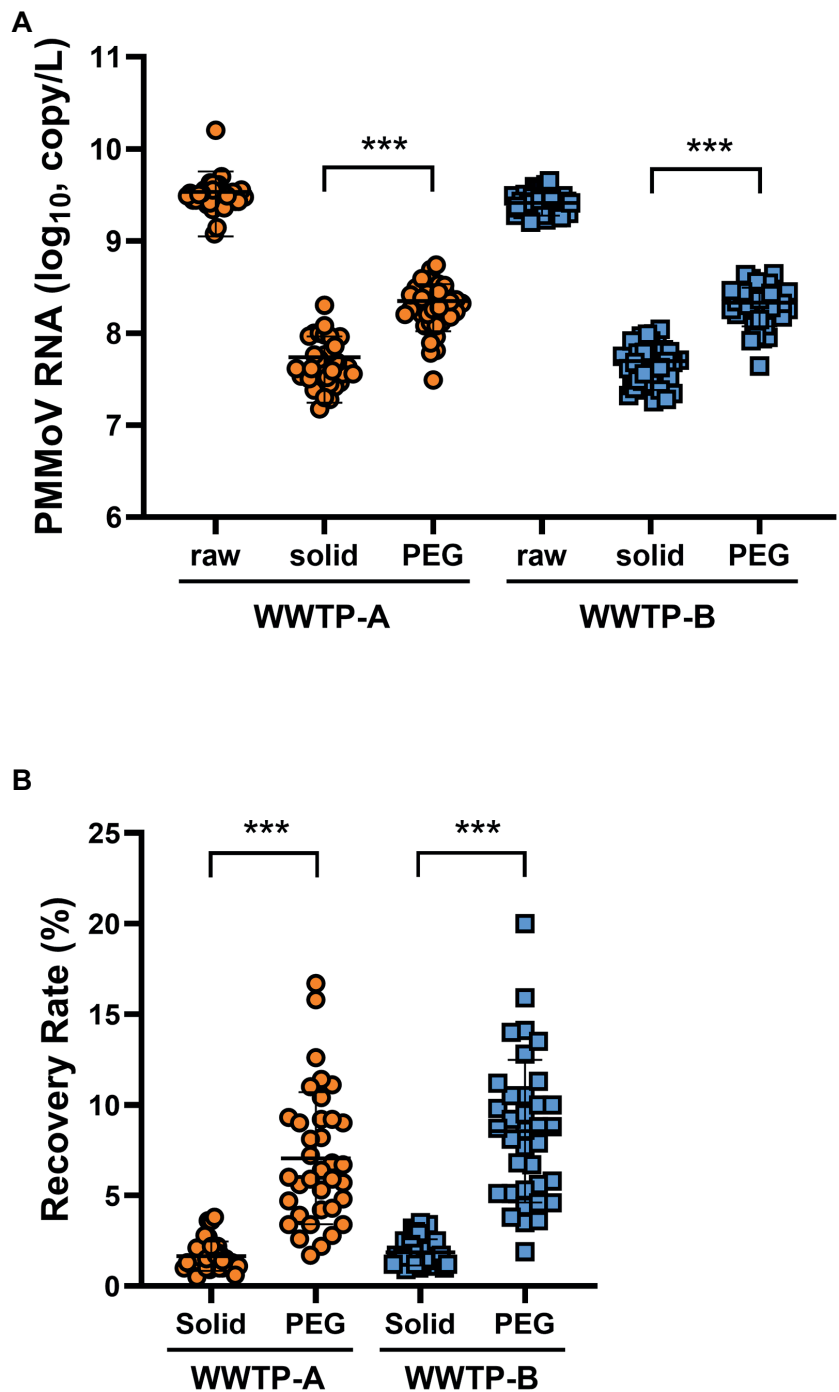
Quantification of pepper mild mottle virus (PMMoV) RNA. **(A)** RNA samples from wastewater treatment plant (WWTP)-A (orange circles) and WWTP-B (blue squares) were quantified for PMMoV RNA copy number *via* RT-qPCR. **(B)** Recovery rate (%) of concentrated samples calculated from PMMoV RNA copy number. Raw: unconcentrated raw wastewater; solid: solid fraction; PEG: PEG-precipitated fraction. Bars indicate the mean ± SD (*n* = 36). Statistical significance was calculated using the Mann–Whitney U test. ^***^p < 0.001.

**Figure 2:**
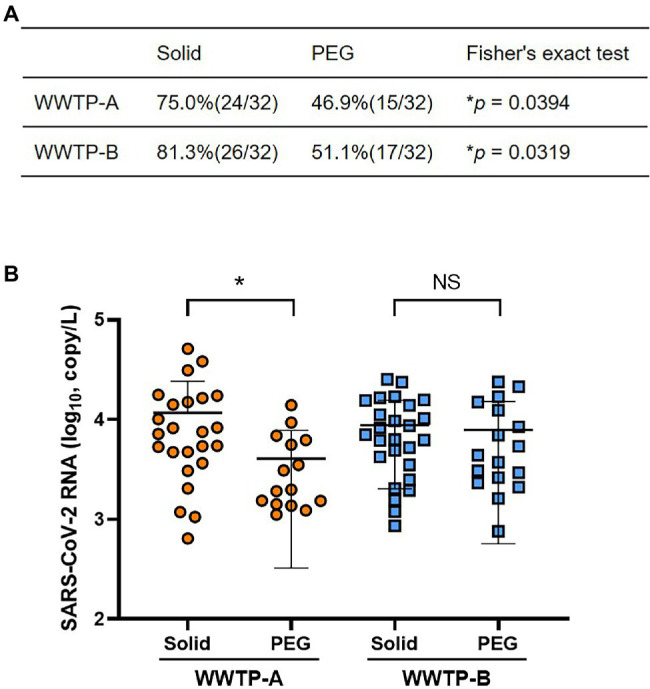
Detection of SARS-CoV-2 RNA in the solid and PEG-precipitated fractions. **(A)** Positivity rate of SARS-CoV-2 RNA in the solid and PEG-precipitated fractions. Values of *p* were calculated using Fisher’s extract test. **(B)** The SARS-CoV-2 RNA copy number of positive samples in solid and PEG-precipitated fractions from WWTP-A (orange circles) and WWTP-B (blue squares) were quantified using RT-qPCR. Bars indicate the mean ± SD. Statistical significance was calculated using the Mann–Whitney U test. ^*^p < 0.05, NS, not significant.

The PMMoV was used as a control for the RNA extraction process. The PMMoV RNA copy numbers extracted from the non-enriched (raw), solid, and PEG-precipitated samples were in the range 1.2 × 10^9^–1.6 × 10^10^, 1.5 × 10^7^–2.0 × 10^8^, and 3.1 × 10^7^–5.5 × 10^8^ copies/L, respectively (Figure 1A). The recovery rates calculated from the PMMoV RNA copy numbers of solid and PEG-precipitated samples were in the range 0.5%–3.8% and 1.7%–20% (Figure 1B). The RNA copy number and recovery rate of PMMoV RNA in PEG-precipitated fraction were significantly higher than in solid fraction. The detection frequency of SARS-CoV-2 RNA in solid samples was significantly higher than that in PEG-precipitated samples in both WWTP-A and WWTP-B (Figure 2A). SARS-CoV-2 RNA copy numbers in the solid fraction and PEG-precipitated fraction were in the range 3.1 × 10^2^–3.8 × 10^4^ and 7.6 × 10^2^–2.4 × 10^4^, respectively (Figure 2B). While SARS-CoV-2 RNA copy numbers in the solid fraction samples were significantly higher than in the PEG-precipitated fraction from WWTP-A, no significant difference was observed between both the fraction from WWTP-B.

### Association Between RNA Copy Number and Infected Case Number

**Figure 3:**
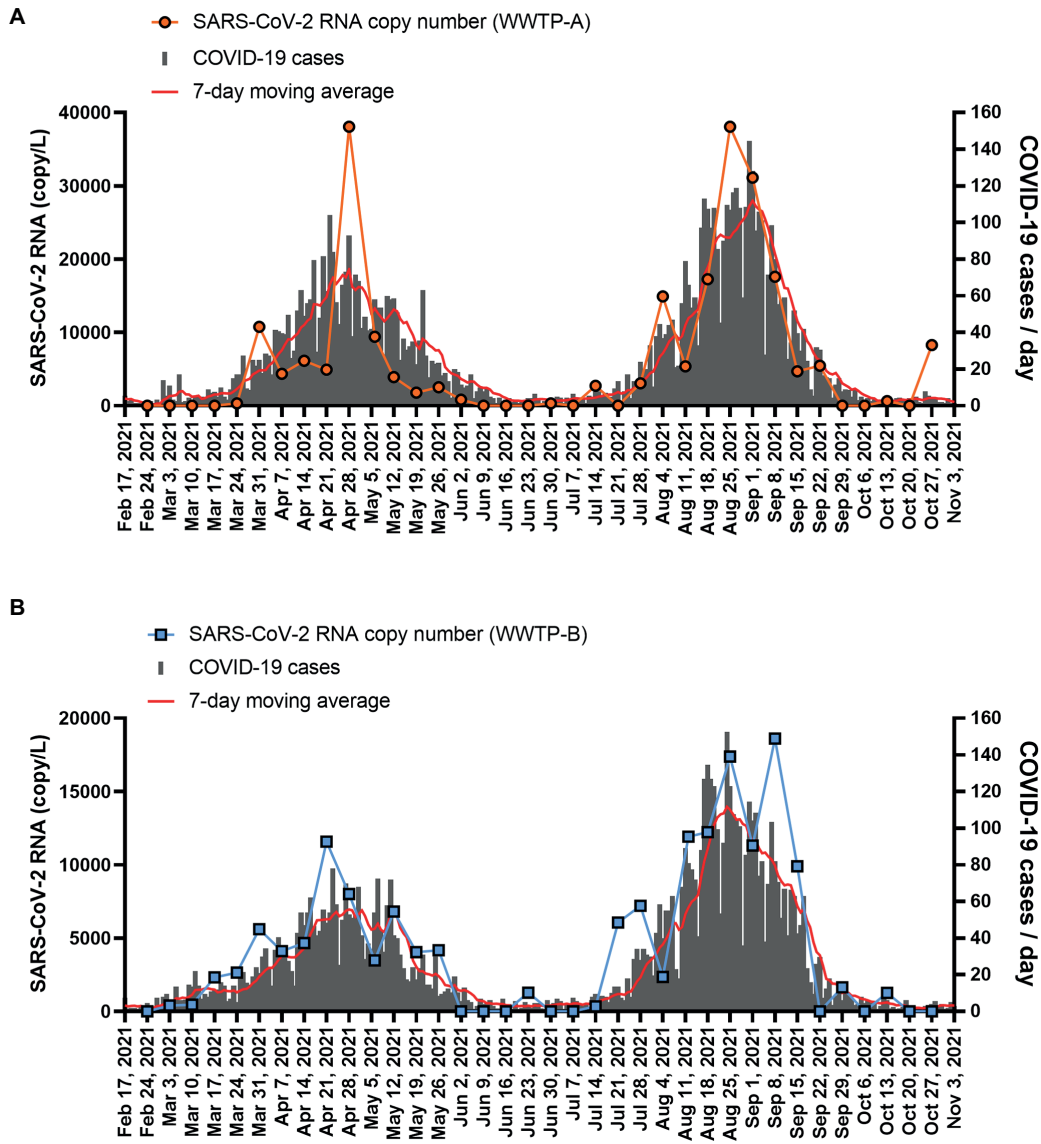
SARS-CoV-2 RNA copy number in the solid fraction and COVID-19 case numbers. The SARS-CoV-2 copy numbers in the solid fraction from **(A)** WWTP-A (orange circle) and **(B)** WWTP-B (blue squares) are plotted. The number of new COVID-19 cases per day in WWTP basin is indicated by the gray bars, and the seven-day moving average is indicated by the red line.

**Figure 4:**
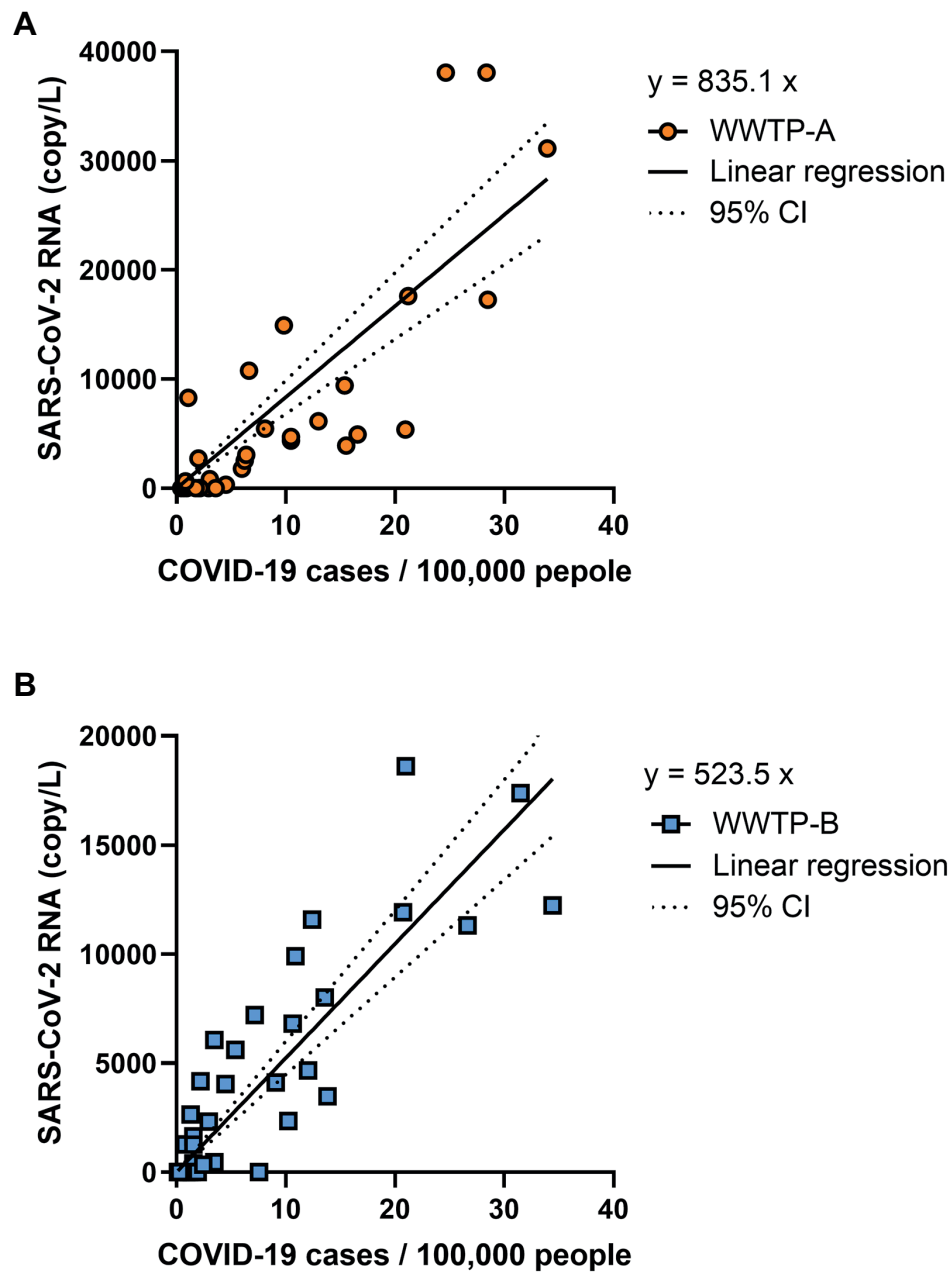
Correlation of COVID-19 cases and SARS-CoV-2 copy number. The daily reported COVID-19 cases per 100,000 people and SARS-CoV-2 RNA copy number in WWTP-A **(A)** and WWTP-B **(B)** were analyzed using linear regression. Lines indicate the linear regression and dotted lines indicate 95% CI.

The SARS-CoV-2 RNA copy number in the solid fraction was highly correlated with the number of COVID-19 cases reported between 24 February 2021 and 27 October 2021 (WWTP-A: *r* = 0.8205, *p* < 0.001; WWTP-B: *r* = 0.8482, *p* < 0.001; Figure 3). While SARS-CoV-2 RNA in the PEG-precipitated fraction and unconcentrated raw samples was significantly correlated with the number of COVID-19 cases between 24 February 2021 and 27 October 2021 (PEG: WWTP-A: *r* = 0.6237, *p* < 0.001; WWTP-B: *r* = 0.7803, *p* < 0.001, and Raw: WWTP-A: *r* = 0.6285, *p* < 0.001; WWTP-B: *r* = 0.4517, *p* = 0.0057; Supplementary Figure S2), the correlation between RNA copy number and COVID-19 cases was lower than that in the solid fraction. The relationships between SARS-CoV-2 RNA and COVID-19 cases in WWTPs basin were evaluated using linear regression analysis (Figure 4). In this study, detection limit of SARS-CoV-2 RNA by RT-PCR was 2.5 copy/reaction, which is calculated to be 625 copy/L in the solid fraction. The limit of capturing COVID-19 cases per 100,000 people calculated using slope were 0.75 cases in WWTP-A and 1.20 cases in WWTP-B, respectively. When the effect of rain on viral RNA concentrations was evaluated, no inverse correlation was found between the amount of rainfall and SARS-CoV-2 RNA in solid fraction (WWTP-A: *r* = 0.1973, *p* = 0.2047; WWTP-B: *r* = 0.1539, *p* = 0.3701; Supplementary Figures S3A,B). Likewise, no inverse correlation between the amount of influent flow and RNA concentration was observed (WWTP-A: *r* = 0.4088, *p* = 0.0133; WWTP-B: *r* = 0.1652, *p* = 0.3356; Supplementary Figures S3C,D).

## Discussion

In this study, SARS-CoV-2 RNA was detected at higher levels in the solid fractions than in the PEG-precipitated fractions, and the RNA copy numbers reflected the infection trend numbers during the fourth and fifth pandemic waves in Kobe, Japan.

The fraction in which higher RNA copy numbers were measured differed between non-enveloped PMMoV and enveloped SARS-CoV-2; PMMoV RNA was higher in the PEG-precipitated liquid fraction, whereas SARS-CoV-2 RNA was higher in the solid fraction. This difference may be related to the structure of the viruses. In one study, up to 26% of enveloped viruses, such as mouse hepatitis virus and bacteriophage φ6, were bound to the solid fraction, whereas only 6% of non-enveloped viruses, such as bacteriophages MS2 and T3, were similarly bound in wastewater samples (Ye et al., 2016). The results of our study are consistent with those of a previous study that reported the detection efficiency of PMMoV in the PEG-precipitated liquid fraction significantly higher than in the solid fraction (Graham et al., 2021). Similar to our findings, RNA extraction from the solid fraction was better than that from liquid fraction for detecting SARS-CoV-2 in previous reports comparing enrichment methods, including pelleted solid fraction, PEG precipitation, electronegative membrane adsorption, and ultrafiltration (Kitamura et al., 2021; Westhaus et al., 2021). In contrast, another study reported that approximately 90% of the SARS-CoV-2 RNA was present in the liquid phase of the influent wastewater compared to the RNA sorbed on the influent solids (Weidhaas et al., 2021). Tomasino et al. (2021) reported that no significant differences were observed in Ct values of SARS-CoV-2 RNA between the liquid and solid phases. These differences are thought to be due to the centrifuge conditions for solid collection. In this study, since centrifugal condition described in previous studies (Kitamura et al., 2021; Westhaus et al., 2021) did not completely precipitate the solid, we set a strong centrifugal condition for ease of work and efficient solid recovery. The different solid removal strategies may result in a high or low representation of the virus in the solid fraction. To minimize errors in RT-PCR detection and quantification, it is recommended that SARS-CoV-2 RNA should be concentrated from both liquid and solid phases of wastewater (Ahmed et al., 2022). Furthermore, in this study, SARS-CoV-2 RNA could be detected using a non-enrichment method as a result of the large number of COVID-19 cases. A previous study reported that the RNA copy number of unenriched wastewater correlated with the number of cases in Marseille, France (Wurtz et al., 2021). These results suggest that the wastewater enrichment methods are not always necessary in areas with high number of COVID-19 cases.

Recovery rate using the PMMoV copy number have been conducted for concentration efficiency of wastewater. Previous studies that compared the liquid and solid fraction showed that the detection efficiency of PMMoV ranged 8.0%–30% in the PEG-precipitated liquid fraction and 6.0%–17% in the solid fraction (Graham et al., 2021), and 12%–102% from liquid fractions and 9.4%–62% from solid fractions (Alamin et al., 2022). Our reported recovery values tended to be lower than the range reported in previous studies. The copy numbers of PMMoV RNA were in the range 8.2 × 10^6^–3.1 × 10^8^ copy/L in the liquid fraction and 1.6 × 10^2^–1.0 × 10^7^ copy/L in the solid fraction (Kitamura et al., 2021). Hasing et al. (2021) reported that the copy numbers of PMMoV were median values of 8.98 × 10^6^ (interquartile range, 6.38 × 10^6^–1.20 × 10^7^) copies per 100 ml in the liquid fraction, and 1.71 × 10^6^ (interquartile range, 1.52 × 10^6^–2.58 × 10^6^) copies per 100 ml in the solids. PMMoV copy numbers in our study did not deviate significantly from the ranges in previous studies, suggesting that the process of wastewater concentration had been performed properly.

In our study, collection of wastewater samples was performed by spot grab sampling, which can affect the SARS-CoV-2 RNA concentration because wastewater flow in a WWTP is increased by the rain. Rainfall was expected to have little effect on RNA concentrations as no inverse correlation between amount of rainfall/influent flow and SARS-CoV-2 RNA in solid was observed. Previous studies showed that composite samples, which were collected by flow-weighting for 24 h, were more detectable than grab samples for SARS-CoV-2 RNA in wastewater (Gerrity et al., 2021; Monteiro et al., 2022). However, grab sampling had been performed for SARS-CoV-2 RNA detection (Randazzo et al., 2020; Carrillo-Reyes et al., 2021; Kitamura et al., 2021; Street et al., 2021; Wehrendt et al., 2021), and correlated with COVID-19 cases (Kitamura et al., 2021; Street et al., 2021). While grab samples have the limitation of low sensitivity, they could be a useful sampling method because they have the advantage of being collected quickly, do not require automated equipment, and were able to reflect COVID-19 cases in our study.

Our data showed that SARS-CoV-2 RNA was detected at higher concentrations as the number of COVID-19 cases increased. This result is consistent with that of a previous study in Tokyo, Japan, which compared SARS-CoV-2 RNA levels in the solid fraction of wastewater with the number of COVID-19 cases from June 2020 to August 2020 (Kitamura et al., 2021). The number of SARS-CoV-2 RNA in primary settled solids collected from primary clarifier was correlated with COVID-19 cases in a study conducted in California, United States (Graham et al., 2021; Wolfe et al., 2021). The number of positive COVID-19 cases has also been correlated with SARS-CoV-2 RNA in wastewater in other countries (Medema et al., 2020a; Carrillo-Reyes et al., 2021; Wurtz et al., 2021); however, the results differed on whether the detection of SARS-CoV-2 RNA increased before or coincident with the number of COVID-19 cases. The viral load in wastewater preceded clinical data by 4 days to 2 weeks in some studies (Medema et al., 2020a; Randazzo et al., 2020; Trottier et al., 2020; Claro et al., 2021; Wu et al., 2022), whereas no time difference was reported in other studies (Peccia et al., 2020; Kitamura et al., 2021). Our data also showed no time difference when correlating the number of COVID-19 cases and SARS-CoV-2 RNA levels. In addition, a study performed daily composite collection of wastewater and reported that although the trend in SARS-CoV-2 RNA levels preceded the number of cases during the first infection wave in France, both measures followed a similar curve in the second infection wave (Wurtz et al., 2021). The difference in the trends of the two waves was probably due to differences in the duration of recognition of the number of cases, that is, in the early stages of a pandemic it is difficult to determine the number of cases as reporting is relatively late, which suggests that the detection of viral RNA precedes case load. In Kobe City, because of active investigation of close contacts and efforts to ascertain the number of infected people, the time difference in reporting the number of cases may be reduced; thus, the viral RNA level and the number of cases correlate without an apparent time difference. In addition, the detection of SARS-CoV-2 RNA from WWTP-B occurred earlier than in WWTP-A. In fact, the number of COVID-19 cases in the WWTP-B basin tended to peak earlier than in WWTP-A.

Linear regression analysis between SARS-CoV-2 RNA copy number and COVID-19 cases showed that the limit of capturing COVID-19 cases per 100,000 people was 0.75 cases in WWTP-A and 1.20 cases in WWTP-B, respectively. SARS-CoV-2 RNA in wastewater was quantifiable in some WWTP basins with daily positive test rates of less than 1 per 10,000 people (Wilder et al., 2021). To detect of SARS-CoV-2, approximately 0.12% and 0.09% of the total population in the WWTP basin area were required to be assessed (Chavarria-Miró et al., 2021). SARS-CoV-2 in wastewater samples collected from five WWTPs in Japan was more likely to be detected when there were more than 10 confirmed cases of COVID-19 per 100,000 people in the basin area, but it was detectable in wastewater even before the number of cases reached 1 per 100,000 people (Hata et al., 2021). The current study found that the capturing COVID-19 cases was equal to or higher than in previous studies. In this study, RNA concentration was detectable in the range 7.6 × 10^2^–2.4 × 10^4^ copy/L when COVID-19 cases per 100,000 people were in the range 0.77–34.4. Previous studies showed that SARS-CoV-2 RNA was detected in the following ranges at the following COVID-19 case rates (per 100,000 people); 1.7 × 10^3^–3.8 × 10^5^ copy/L at 4.8–57.3 cases (D’Aoust et al., 2021), 1.2 × 10^1^–2.2 × 10^3^ copy/L at 0.1–100 cases (Medema et al., 2020a), and 3.0 × 10^3^–2.0 × 10^4^ copy/L at 30–174 cases (Westhaus et al., 2021). Medema et al. (2020b) reported a simulation model of the number of COVID-19 infected people in the population and concentration of SARS-CoV-2 RNA in sewage, and estimated that RNA copy number was approximately 10^2^–10^5^ copy/L at 10–100 cases per 100,000 people. Our results are consistent with these previous studies, suggesting that RNA concentration reflects COVID-19 case numbers.

In Japan, the fourth and fifth waves of SARS-CoV-2 RNA were useful predictions of manifesting COVID-19 cases. The present data indicate that the detection of SARS-CoV-2 RNA in sewage can be used to monitor and predict trends in SARS-CoV-2 infections. This monitoring may provide valuable data even when the number of patients diagnosed with COVID-19 at clinical sites becomes low owing to mass vaccination. In the mass vaccination era, the number of asymptomatic cases is expected to increase, making it more difficult to determine the actual number of cases in the community. The usefulness of wastewater-based epidemiology, which can determine the number of both infected and asymptomatic persons in a community, will increase in the future. These results show the potential of using sewage monitoring, such as RNA levels, in public health, including responding to and the issuing of health warnings within sewerage basins.

## Data Availability Statement

The original contributions presented in the study are included in the article/Supplementary Material, and further inquiries can be directed to the corresponding authors.

## Author Contributions

YT, TM, and TI: conceptualization. YT and EI: methodology and writing—original draft preparation. NO and TM: resources. YT, EI, SM, AM, RN, and NN: investigation. YT: formal analysis and funding acquisition. RN and TI: supervision. YT, EI, SM, AM, RN, NN, NO, TM, and TI: writing—review and editing. All authors contributed to the article and approved the submitted version.

## Funding

This study was partially supported by a grant from the Daido Life Welfare Foundation for Regional Health and Welfare Research from 2021 to YT.

## Conflict of Interest

The authors declare that the research was conducted in the absence of any commercial or financial relationships that could be construed as a potential conflict of interest.

## Publisher’s Note

All claims expressed in this article are solely those of the authors and do not necessarily represent those of their affiliated organizations, or those of the publisher, the editors and the reviewers. Any product that may be evaluated in this article, or claim that may be made by its manufacturer, is not guaranteed or endorsed by the publisher.

## Supplementary Material

Supplementary Figure S1 Detection limit of SARS-CoV-2 RNA using RT-qPCR. Each copy of the standard SARS-CoV-2 RNA (Ct value) was confirmed using RT-qPCR measurement. The slope, intercept, and coefficient of determination (*R^2^*) values were calculated using linear regression.

Supplementary Figure S2 SARS-CoV-2 RNA copy number in the PEG-precipitated or unconcentrated sample and COVID-19 case numbers. The SARS-CoV-2 copy numbers in the liquid fraction concentrated using PEG precipitation **(A,B)** or unconcentrated raw sample **(C,D)** from WWTP-A (**A,C**; orange circles) and WWTP-B (**B,D**; blue squares) are plotted. The number of new COVID-19 cases per day in WWTP basin is indicated by the gray bars, and the seven-day moving average is indicated by the red line.

Supplementary Figure S3 The amount of rainfall and SARS-CoV-2 RNA copy number in the solid fraction. The SARS-CoV-2 RNA copy number in the solid fraction from WWTP-A (**A,C**; orange circles) and WWTP-B (**B,D**; blue squares) are plotted. The amount of rainfall in each WWTPs is indicated by the purple bars **(A,B)** and the amount of influent flow is indicated by the black line **(C,D)**.
