# Supplementary material for: SARS-CoV-2 RNA in Wastewater Was Highly Correlated With the Number of COVID-19 Cases During the Fourth and Fifth Pandemic Wave in Kobe City, Japan
Source: Front Microbiol. 2022 Jun 9;13:892447. doi: 10.3389/fmicb.2022.892447 (PMC9223763; doi:10.3389/fmicb.2022.892447)
Supplement: Supplementary Figure S3 — The amount of rainfall and SARS-CoV-2 RNA copy number in the solid fraction. The SARS-CoV-2 RNA copy number in the solid fraction from WWTP-A (A,C; orange circles) and WWTP-B (B,D; blue squares) are plotted. The amount of rainfall in each WWTPs is indicated by the purple bars (A,B) and the amount of influent flow is indicated by the black line (C,D). [file Presentation_1.PPTX]

## Slide 1
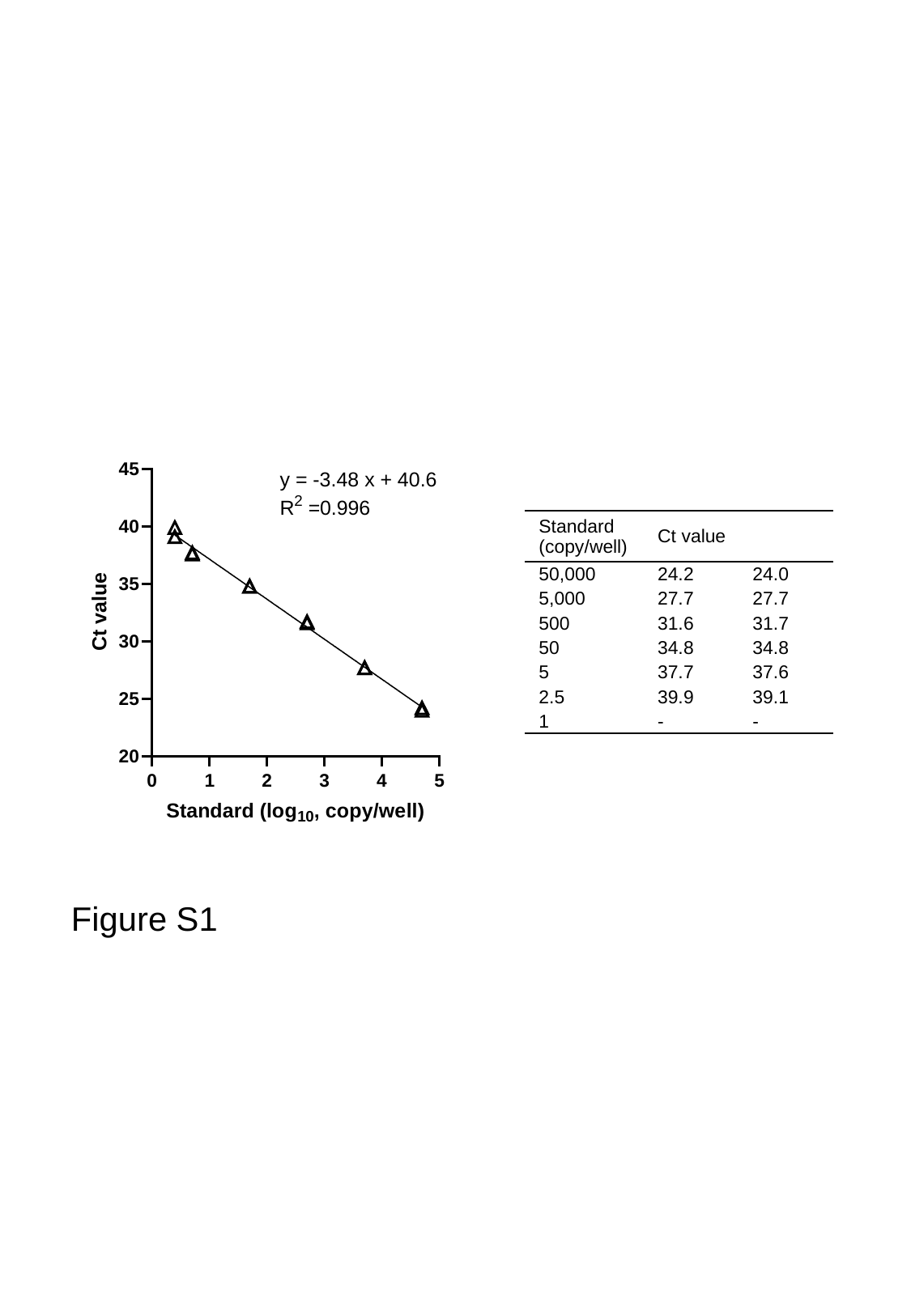

| Standard (copy/well) | Ct value | |
| --- | --- | --- |
| 50,000 | 24.2 | 24.0 |
| 5,000 | 27.7 | 27.7 |
| 500 | 31.6 | 31.7 |
| 50 | 34.8 | 34.8 |
| 5 | 37.7 | 37.6 |
| 2.5 | 39.9 | 39.1 |
| 1 | - | - |
Figure S1

## Slide 2
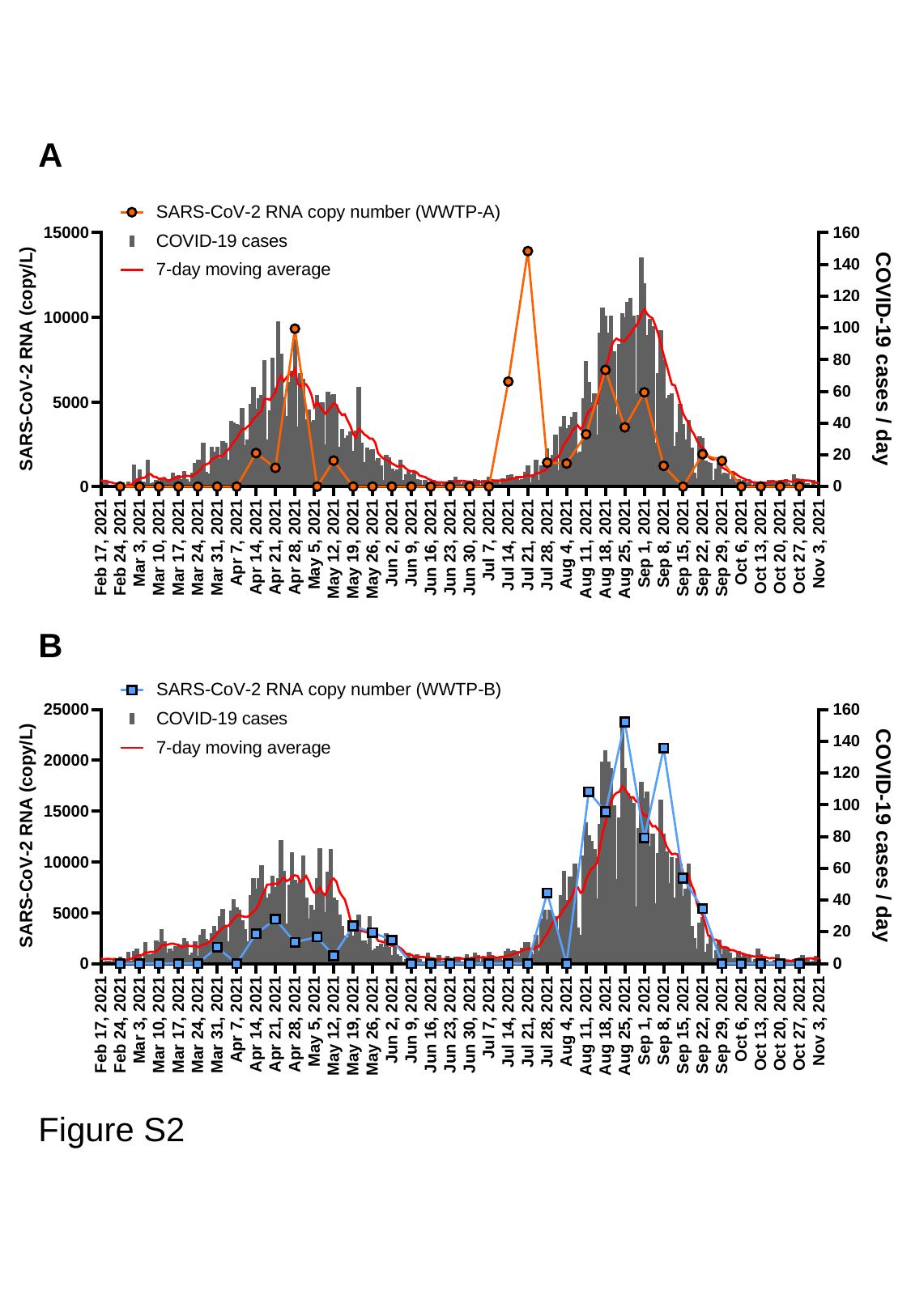

A
B
Figure S2

## Slide 3
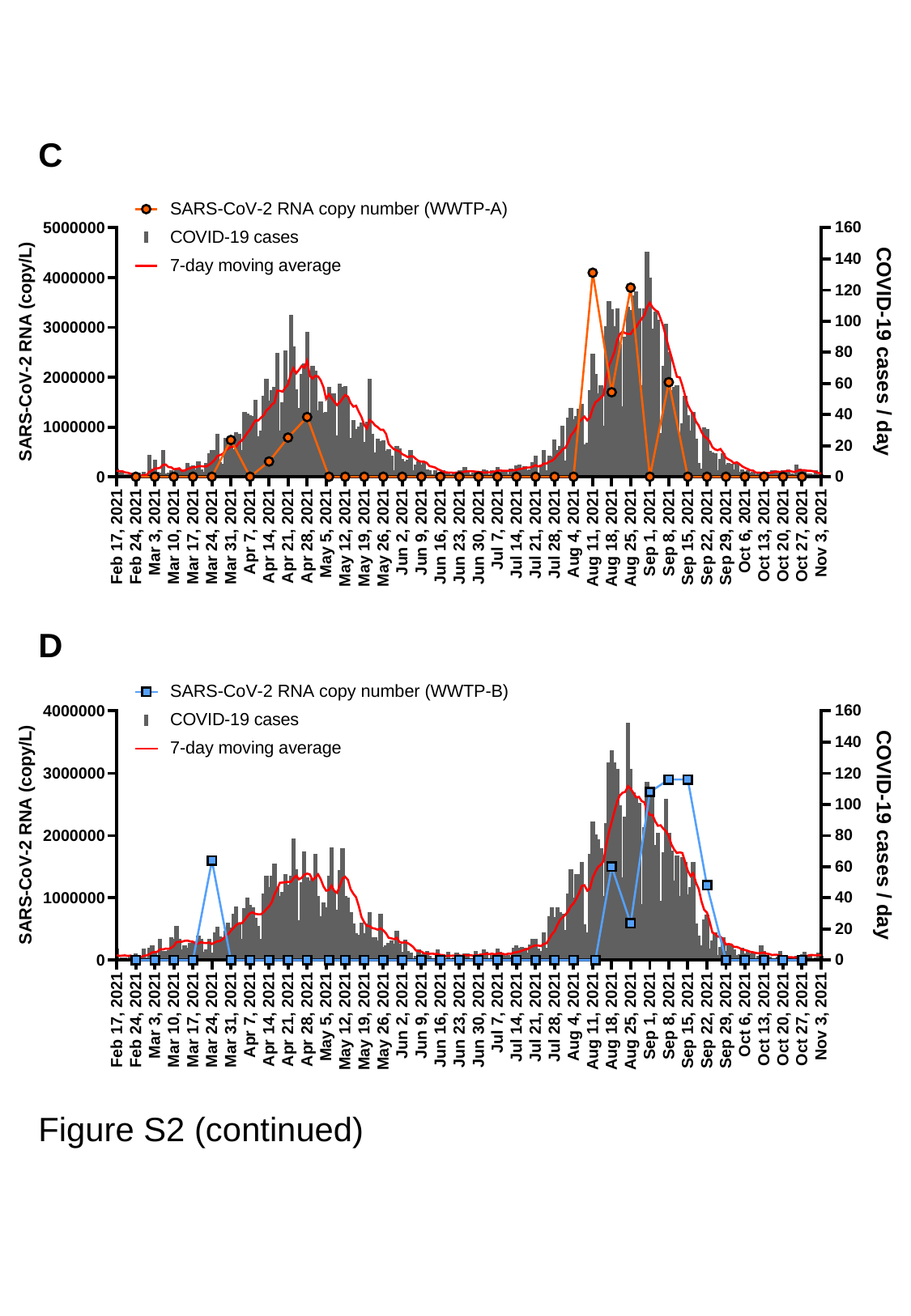

C
D
Figure S2 (continued)

## Slide 4
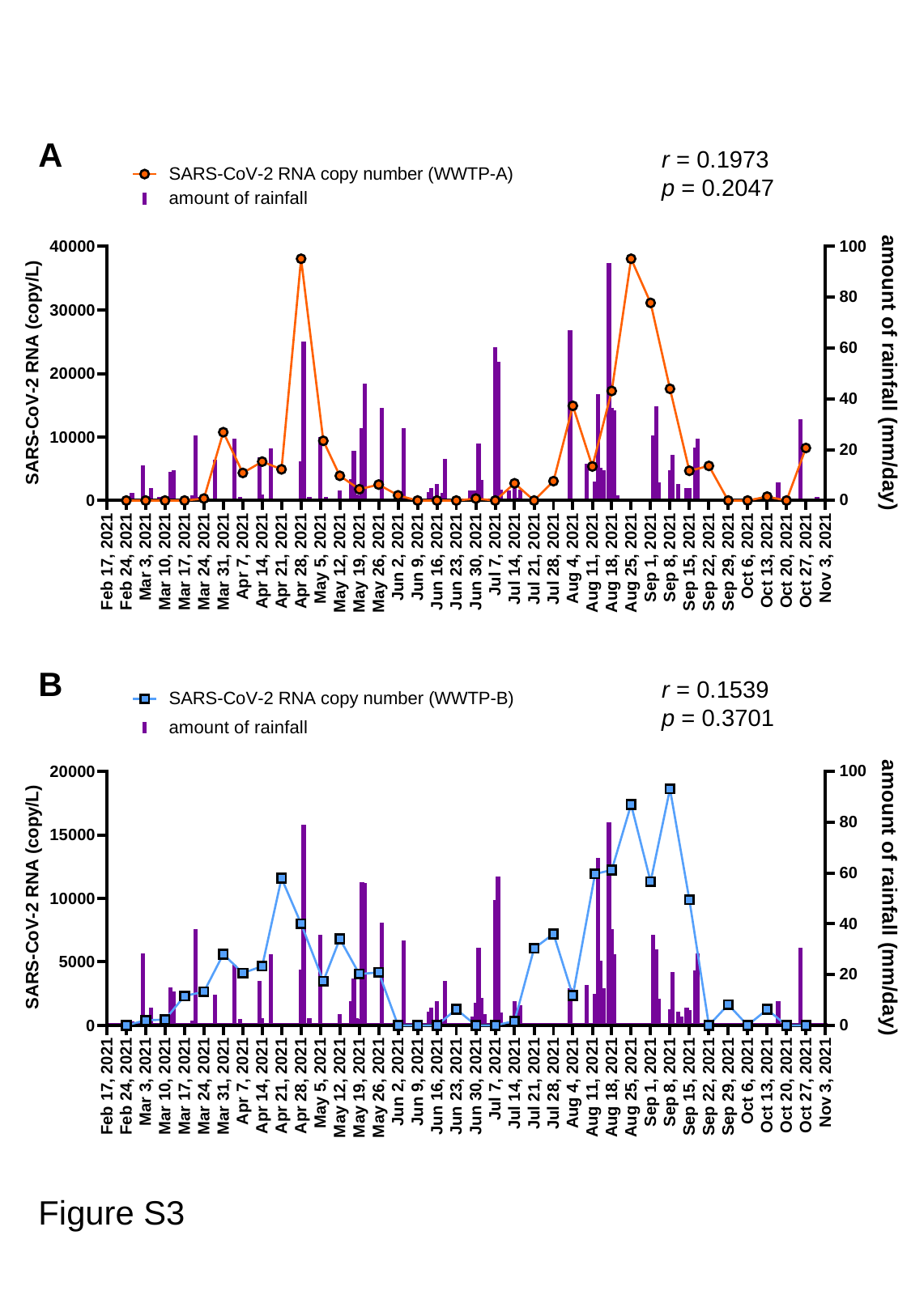

A
r = 0.1973
p = 0.2047
B
r = 0.1539
p = 0.3701
Figure S3

## Slide 5
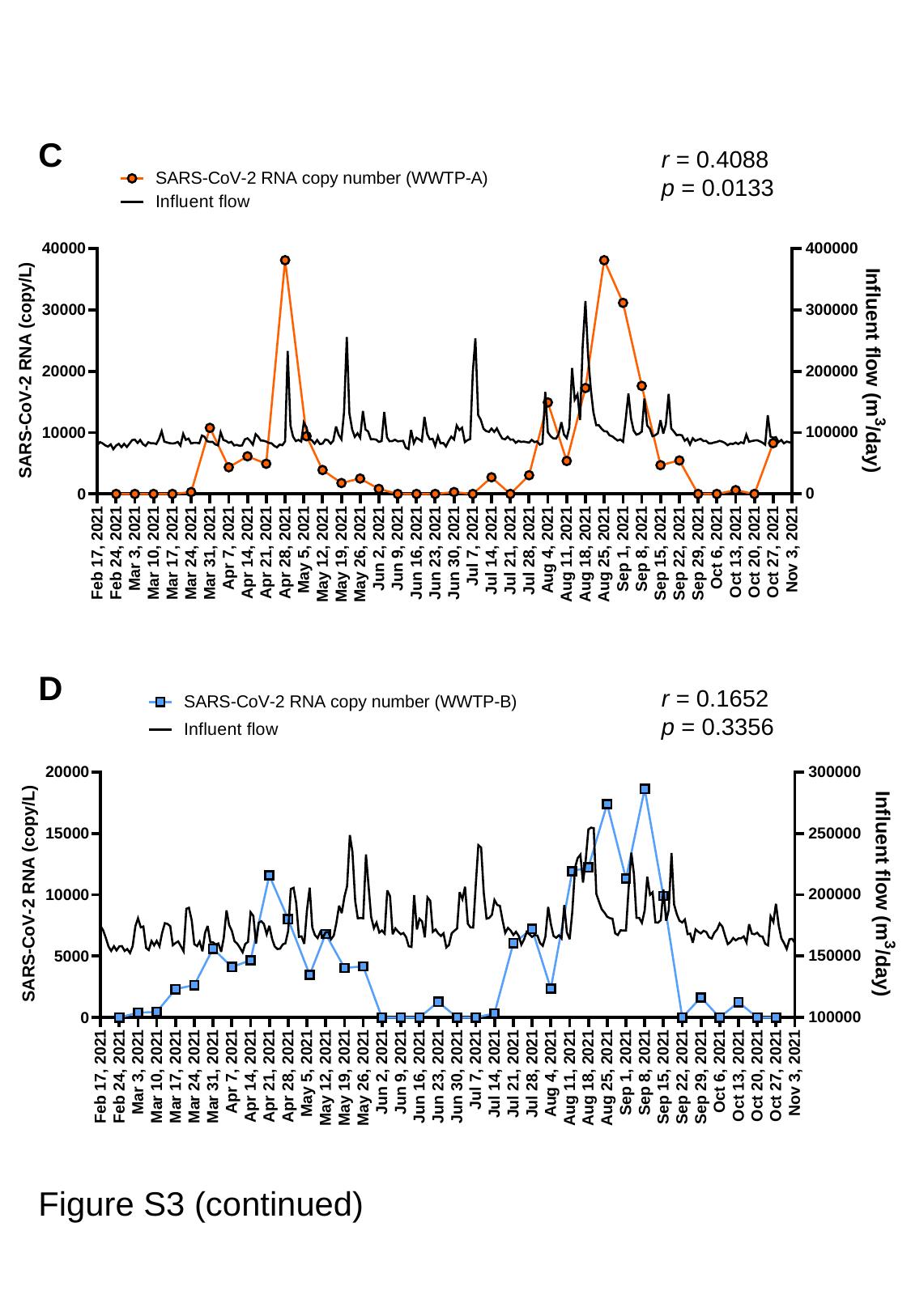

C
r = 0.4088
p = 0.0133
D
r = 0.1652
p = 0.3356
Figure S3 (continued)
